# Supplementary material for: Genome-Wide Identification of the ARF Gene Family in Safflower (Carthamus tinctorius L.) and Their Response Patterns to Exogenous Hormone Treatments
Source: Int J Mol Sci. 2025 Apr 16;26(8):3773. doi: 10.3390/ijms26083773 (PMC12028013; doi:10.3390/ijms26083773)
Supplement: Supplementary file 1 [file ijms-26-03773-s001.zip › Supplementary Figure S2.docx]

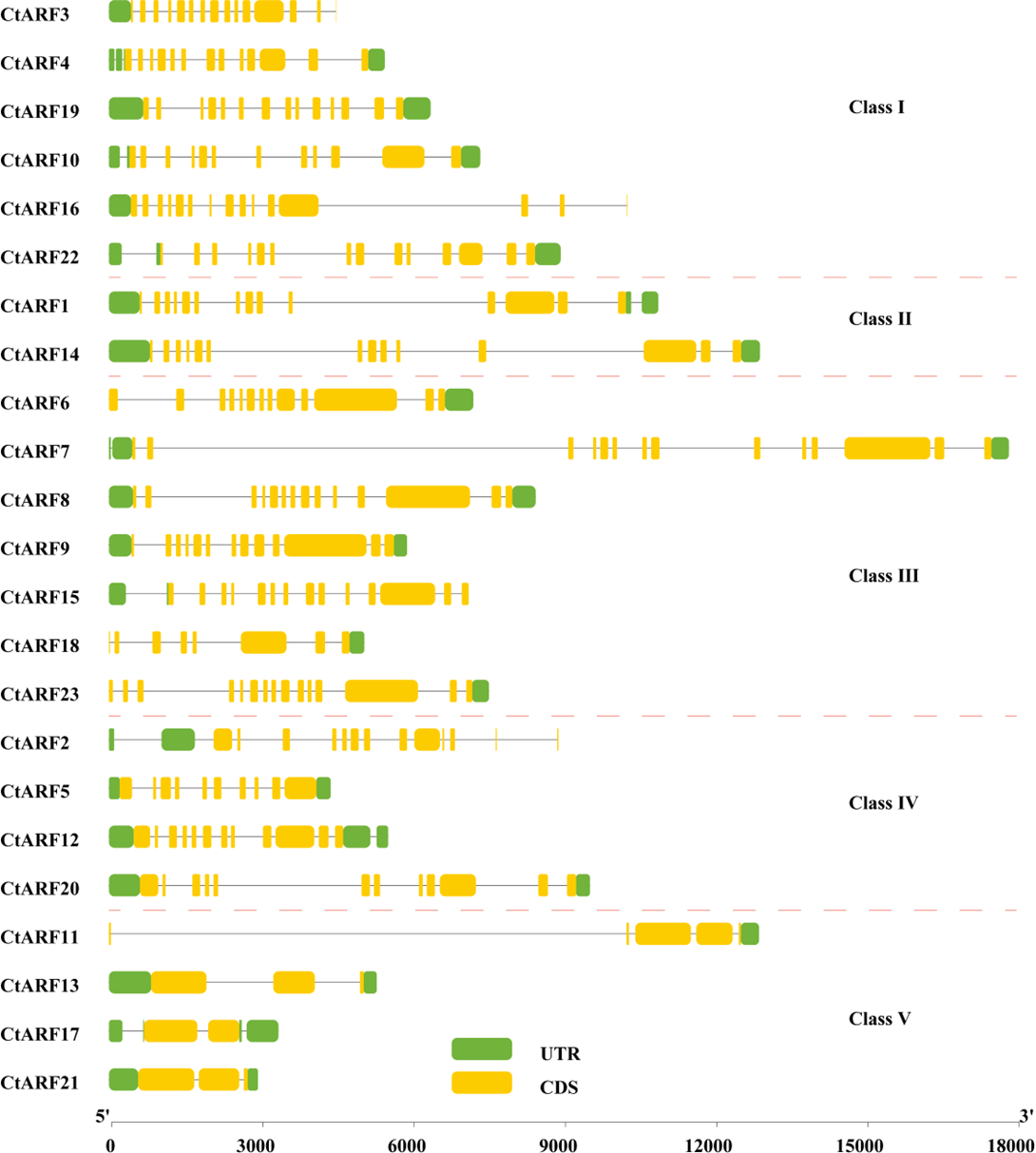


**Supplementary Figure S2. Gene structure analysis of *ARF* genes in safflower. Yellow boxes represent exons, black lines represent introns, and green boxes represent untranslated regions. The UTR refers to the untranslated region, while the CDS refers to the coding sequence.**
