# Supplementary material for: Genome-Wide Identification of the ARF Gene Family in Safflower (Carthamus tinctorius L.) and Their Response Patterns to Exogenous Hormone Treatments
Source: Int J Mol Sci. 2025 Apr 16;26(8):3773. doi: 10.3390/ijms26083773 (PMC12028013; doi:10.3390/ijms26083773)
Supplement: Supplementary file 1 [file ijms-26-03773-s001.zip › Supplementary Figure S4.docx]

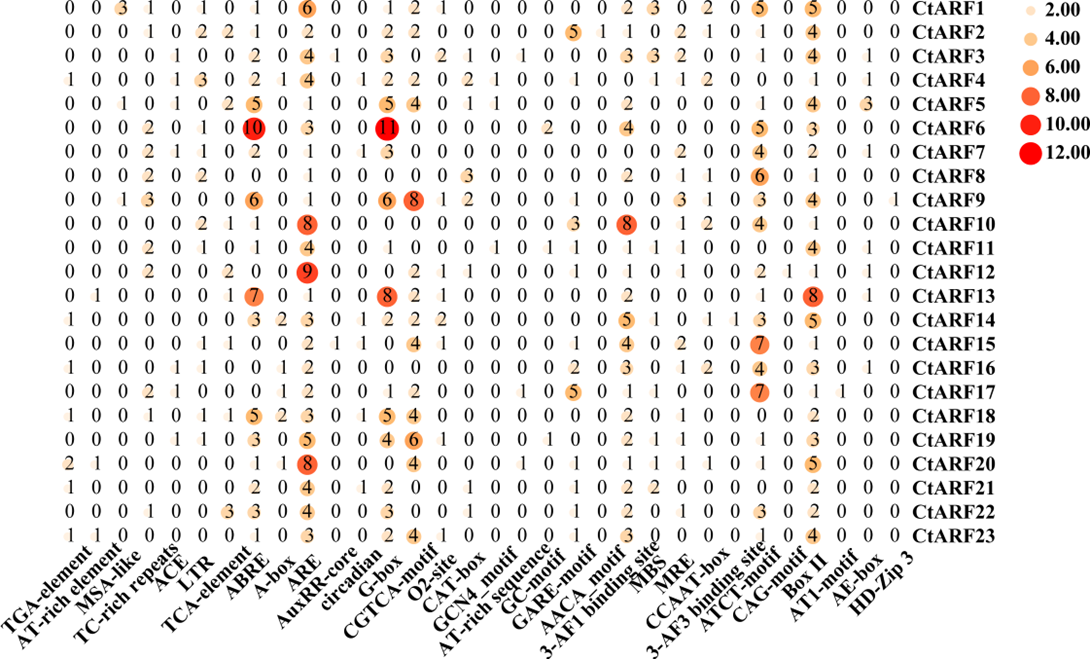


**Supplementary Figure S4. Heatmap analysis of cis-acting elements in the promoter regions of safflower ARF genes. Circles represent the number of cis-acting elements, with larger circles indicating higher numbers.**
