# Supplementary material for: Genome-Wide Identification of the ARF Gene Family in Safflower (Carthamus tinctorius L.) and Their Response Patterns to Exogenous Hormone Treatments
Source: Int J Mol Sci. 2025 Apr 16;26(8):3773. doi: 10.3390/ijms26083773 (PMC12028013; doi:10.3390/ijms26083773)
Supplement: Supplementary file 1 [file ijms-26-03773-s001.zip › Supplementary Figure S5.docx]

**
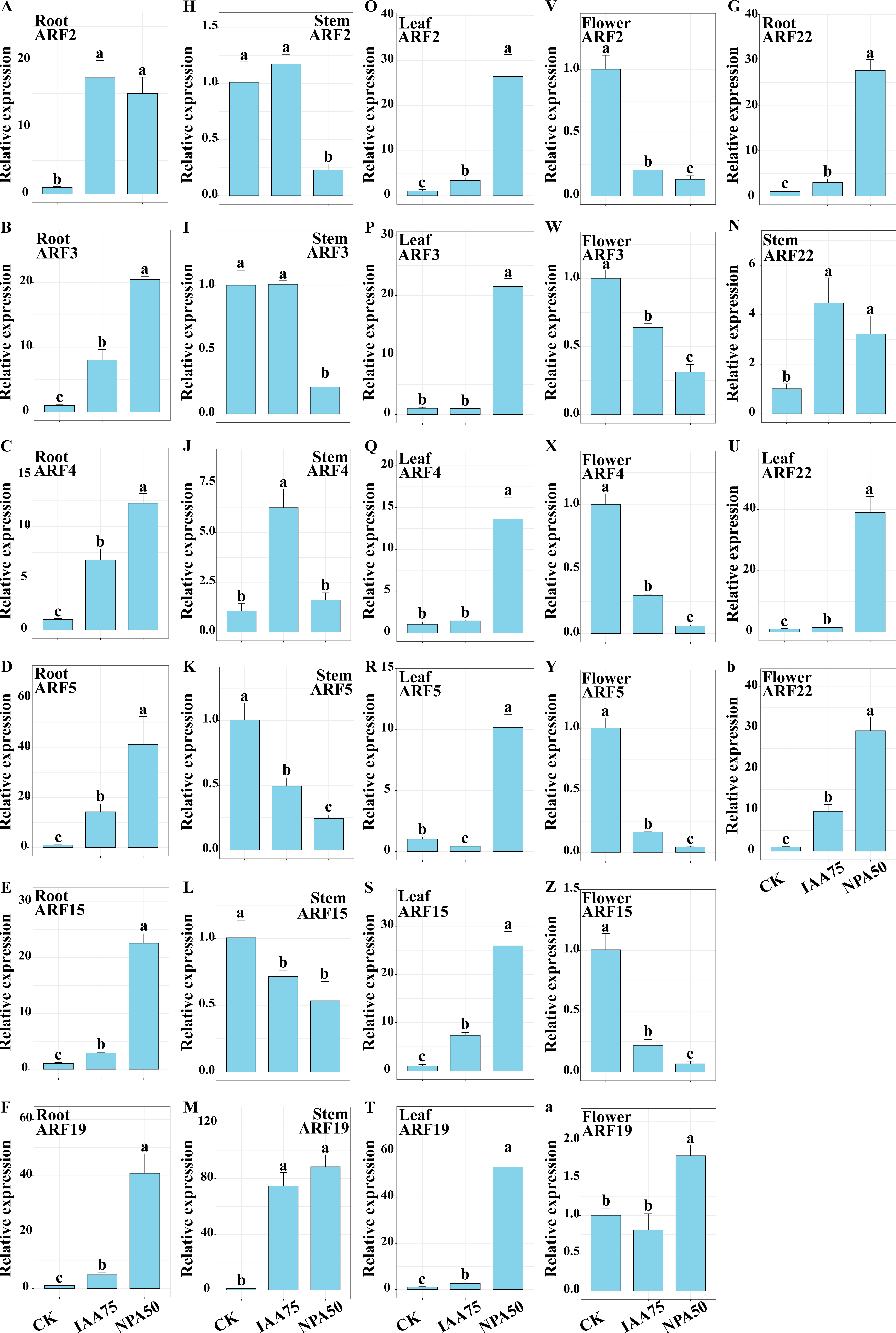
**

**Supplementary Figure S5. Determination of the relative expression levels of some ARF family members in different organs of safflower (WS) after exogenous hormone treatment (IAA and NPA). Different lowercase letters indicate significant differences.**
