# Supplementary figures and images for: Genome-Wide Identification of the ARF Gene Family in Safflower (Carthamus tinctorius L.) and Their Response Patterns to Exogenous Hormone Treatments
Source: Int J Mol Sci. 2025 Apr 16;26(8):3773. doi: 10.3390/ijms26083773 (PMC12028013; doi:10.3390/ijms26083773)

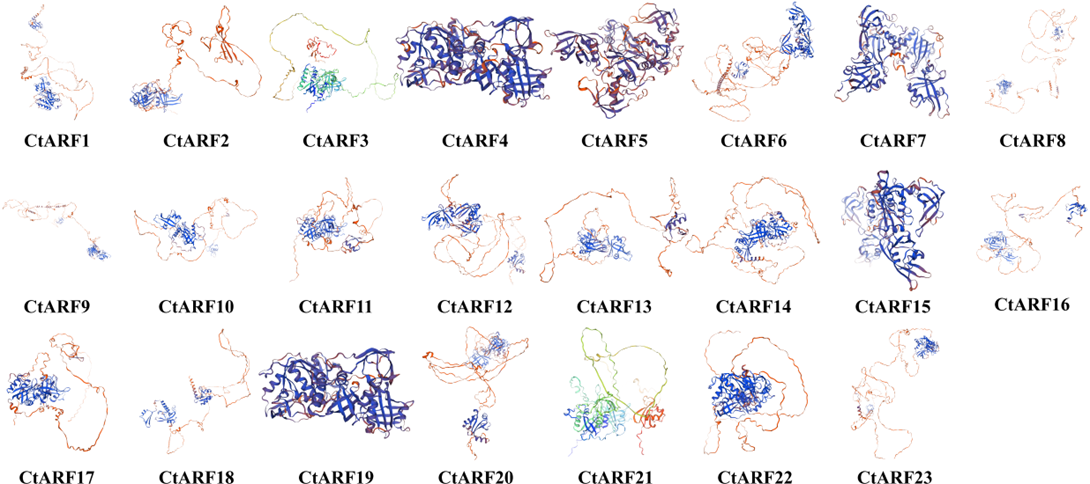


**Supplementary Figure S3. Prediction of the tertiary structure of ARF proteins in safflower.**

Supplement: Supplementary file 1 [file ijms-26-03773-s001.zip › Supplementary Figure S3.docx]

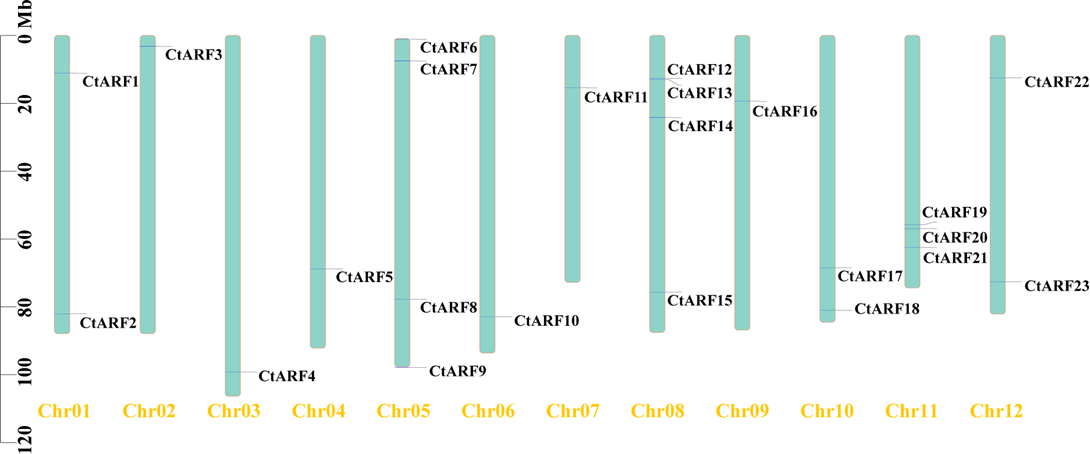


**Supplementary Figure S6. Chromosomal distribution of CtARFs.**

Supplement: Supplementary file 1 [file ijms-26-03773-s001.zip › Supplementary Figure S6.docx]
